# Pelagic seabird density and vulnerability in the Gulf of Mexico to oiling from the *Deepwater Horizon*/MC-252 spill

**DOI:** 10.1007/s10661-019-7921-2

**Published:** 2020-03-17

**Authors:** J. C. Haney, J. M. Hemming, P. Tuttle

**Affiliations:** 1 Terra Mar Applied Sciences, LLC, 1370 Tewkesbury Place NW, Washington, DC 20012 USA; 2 U.S. Fish and Wildlife Service, 341 Greeno Rd., Suite A, Fairhope, AL 36532 USA

**Keywords:** Marine birds, *Deepwater Horizon* blow-out, Gulf of Mexico, Natural resource damage assessment

## Abstract

Using ship-based surveys, the Natural Resource Damage Assessment (NRDA) Trustees assessed the external oiling of offshore and pelagic marine birds inhabiting the northern Gulf of Mexico (Gulf) in the year following the *Deepwater Horizon* oil spill (DWH spill). Study objectives were to (1) collect data on pelagic seabirds that were visibly oiled, (2) collect data to estimate abundance of seabirds in offshore and pelagic waters, and 3) document the location and condition of any bird carcasses encountered. Methods employed included strip line transects and station point counts. Surveys were conducted within a study area bound by the Texas-Mexico border and the Dry Tortugas of Florida to the south, and the nearshore coastal waters of the northern Gulf of Mexico. A total of 5665 strip line transects and 386 station point-counts of variable duration were collected during the study. More than 23,000 individual seabirds comprising 45 estuarine, coastal, offshore, and pelagic species were tallied. Average daily abundance of seabirds detected varied from a low of approximately 7 birds/day in November 2010 along regions of the mid- and outer continental shelf to a high of more than 580 birds/day in June 2011 within the near-shore, coastal waters of the northern Gulf.

## Introduction

The *Deepwater Horizon* mobile drilling unit exploded, caught fire and sank on April 20, 2010, initiating release of oil into the Gulf of Mexico. This spill continued for 87 days, discharging a total volume of 134 million gallons into the northern Gulf of Mexico, making it the largest accidental marine oil spill ever in the USA. As such, oil spill-related injury to wildlife was of major concern to the public and Natural Resource Damage Assessment Trustees (Trustees)^1^.

To understand the injury to birds offshore, the Trustees examined the distribution and density of birds at sea with aerial surveys (Ford 2010), but this did not always provide important information on oiled or dead birds. Additionally, not all species of birds can be properly identified through aerial surveys. To assist in characterizing injury to birds in the pelagic zone of the Gulf, the Trustees evaluated mortality of birds and also live oiled birds in the offshore and pelagic zones. Pelagic surveys also provided data on the diversity and abundance of seabirds that were exposed to oil, the primary objective of this paper.

## Methods

### Study area

Beginning in early July 2010 just prior to when the *Deepwater Horizon* well was capped, surveys were conducted within a study area bound by a line connecting the Texas-Mexico border and the Dry Tortugas of Florida to the south, and the nearshore coastal waters of the northern Gulf of Mexico. Being that the capping reduced the presence of oil on the Gulf, these surveys were largely conducted after the heaviest surface water oiling had already occurred in the pelagic environment (8 July 2010–17 July 2011). The Gulf of Mexico’s marine environment is distinguished by its low mean tidal ranges and weak non-storm waves in coastal offshore waters (Conner et al. 1989; Jackson et al. 2002; Morton et al. 2005), and the large clock-wise rotating Loop Current and its associated frontal and detached eddies dominating in deeper pelagic waters (Sturges and Leben 2000; Liu et al. 2011).

### Overall sampling design

Ships conducted survey sampling on a set route among stations that provided for observing both sitting and flying birds on strip transects between those stations or, in the case of marine mammal surveys, on the same transects used to monitor mammals. Observers collected transect data only while the ship was in transit. However, when research vessels were stopped at sampling stations, bird observers also performed point count surveys. Research vessels were mostly those operated by the National Oceanic and Atmospheric Administration (NOAA); however, one privately chartered oil spill response vessel, one University National Oceanographic System vessel, and one US Coast Guard vessel were also used.

For strip transect surveys, birds occurring within 300 m abeam of the ship were recorded along a distance traveled. Transect surveys were performed when the ship was underway on a straight course at a constant speed. Strip transect surveys were conducted from a viewing platform, generally the wing bridge or flying bridge, that provided an unobstructed view of the water from heights ranging from 10 to 15 m above the sea surface. New transects and new point count surveys arose from any temporary suspension of sampling as the result of (1) ship speed falling below about 11 km h^−1^; (2) other modifications to ship operations, including substantial course changes; and (3) temporary pauses in order to record location or environmental data. Because ship operations were not always predictable, some transects might be less than 10 min duration. Some transects could be conducted in a continuous fashion whereas others were interrupted (resulting in coverage gaps), all depending on the ship operations described just above, including the breaks necessary to rest observers.

All sitting and flying seabirds within a 90° arc (between the bow and abeam) within 300 m of one side of the ship were considered “inside the transect” and relevant to this study for calculating a standardized density estimate. Density estimates were collected and binned within temporal windows of no more than 10 min duration. To account for variable detectability as a function of distance, the distance from the ship’s beam to individual birds was also estimated (e.g., 80 m, 120 m) using a hand-held rangefinder. Birds detected within 100 m of the ship were observed with greatest priority for determining oil exposure. In addition, birds observed within the transect width, but more than 300 m ahead of the ship, and that dove or took flight from the transect, were recorded for purposes of density estimates if it appeared that their behavior occurred in response to the ship.

Track lines and locations were archived using a GPS-recorded position at least every 10 min. All sightings were time-synchronized so as to be comparable with other cruise tracking programs on board the ship. Bird sightings were recorded on paper, on tape, or on digital recorders along with the exact time (calibrated to the GPS). Depending on the equipment available, some sightings were thus aligned to a GPS location post hoc. GPS waypoints for the position of the ship were taken for each sighting of an oiled animal.

Point counts were used when the ship was stopped. The same data was collected for these on-station methods as was collected for trip transect surveys, excluding the track line of the ship. For point counts, the sample was the area within a 90° to 360° arc with a radius of 300 m from the observer in which all birds were recorded. Surveys were conducted from an appropriate vantage point when the ship was at a stationary location at sea for at least 30 min. The observer identified birds within an angle arc appropriate for the vantage point (e.g., 90° arc if looking out one side of the vessel, 270° arc off the bowsprit, or 360° from a crow’s nest). All birds within 300 m were counted and identified to species (when possible). All birds within 100 m were observed for oiling. Behavior was also recorded for each bird.

For both transect and point count surveys (Table 1), observers used binoculars with at least 10× magnification to identify live and dead birds, identify the species (or nearest taxon), and to determine the bird’s oiling status (described below). When available, optics (binoculars or camera) were used to determine with confidence the presence or absence of oil on a bird. For live birds with a confirmed oiling status, observers used a range finder to estimate the distance between the bird and the observer. When two observers were present, during periods of high bird density, one individual would focus on population counts while the other focused on confirmed observations of oiling status. However, priority was given to oiling status observations when only a single observer was available.

**Table 1:** Summary of the total numbers, durations, and lengths (in kilometers) of all 300-m, 10-min strip transects and all point counts completed for NRDA Bird Study #6, July 2010 through July 2011

Cruise	Number transects	Total duration of transects (h:min)	Total length of transects (km)	Number of point counts	Total duration of point counts (h:min)
8–12 July 2010	44	7:20	128.8	16	30:27
13–18 July 2010	128	21:20	380.2	4	4:15
26 July-2 August 2010	346	57:40	865.5	1	0:25
2–7 August 2010	140	23:20	380.9	7	6:45
7–21 August 2010	327	56:37	1011.2	26	18:38
21–26 August 2010	35	2:40	119.4	10	47:33
24 August-9 September 2010	374	62:26	1098.3	28	19:29
13–28 September 2010	169	28:50	474.1	41	40:41
15–28 September 2010	289	48:10	840.7	14	7:00
8–18 October 2010	255	42:30	641.1	0	0:00
13–28 October 2010	255	42:40	682.5	29	21:55
21 October-3 November 2010	252	42:00	550.7	0	0:00
2–12 November 2010	205	34:10	515.4	19	12:08
9–19 November 2010	227	37:50	636.5	0	0:00
16–22 November 2010	138	23:00	295.4	17	5:46
3–14 December 2010	212	35:20	508.7	2	0:56
17 Feb-1 March 2011	217	36:10	589.2	41	18:30
10–22 March 2011	295	49:20	857.0	27	14:30
24–27 March 2011	130	21:40	307.7	1	0:10
7–25 April 2011	122	20:20	329.5	41	71:33
24–28 April 2011	114	19:00	292.7	3	1:30
25–30 April 2011	166	27:40	481.0	3	2:15
2–10 May 2011	279	46:30	724.1	2	1:40
13–27 May 2011	464	77:20	1153.7	0	0:00
7–11 June 2011	91	16:53	319.8	5	3:00
23 June-4 July 2011	191	36:00	568.9	41	9:00
6–17 July 2011	200	33:30	569.4	18	3:20
Grand total	5665	950:16	15,322.4	386	341:26

Observer fatigue was addressed by using two observers wherever possible, with each observer counting continuously for 1 h before being replaced by their alternate. In circumstances when only one observer observed and recorded seabird density data, the second observer observed and recorded oiling status data. For ships in which space limited the placement of only a single observer, that observer monitored and recorded for no longer than two continuous hours, with a 30-min rest between additional observations.

### Oiling rate determination

For each bird, critical data included species (or closest taxon), whether the bird was confidently assessed for oiling, and if confidently assessed, the degree of oiling. For birds for which oiling status could not be confidently assessed, these observations were noted for purposes of estimating population sizes, but they did not contribute to the determination of oiling rate. An oiling status was “confidently assessed” only when the observer had undoubtedly observed oil on the bird or had undoubtedly observed the bird to be free of oil. Any birds for which oiling status could not be confidently determined due to issues with distance from observer, sea state, or weather limiting visibility were noted as “undetermined.”

Oiling rates were only estimated when half or more of the bird’s body surface could be clearly observed. Each bird that was clearly observed was assigned an oiling category via team consensus when possible. In addition to oiling rate, bird-oiling levels were categorized as “None,” “Trace” (greater than 0, but < 5% of the bird’s body surface), “Light” (6–20%), “Moderate” (21–40%), and “Heavy” (> 40%).

## Results

**Fig. 1:**
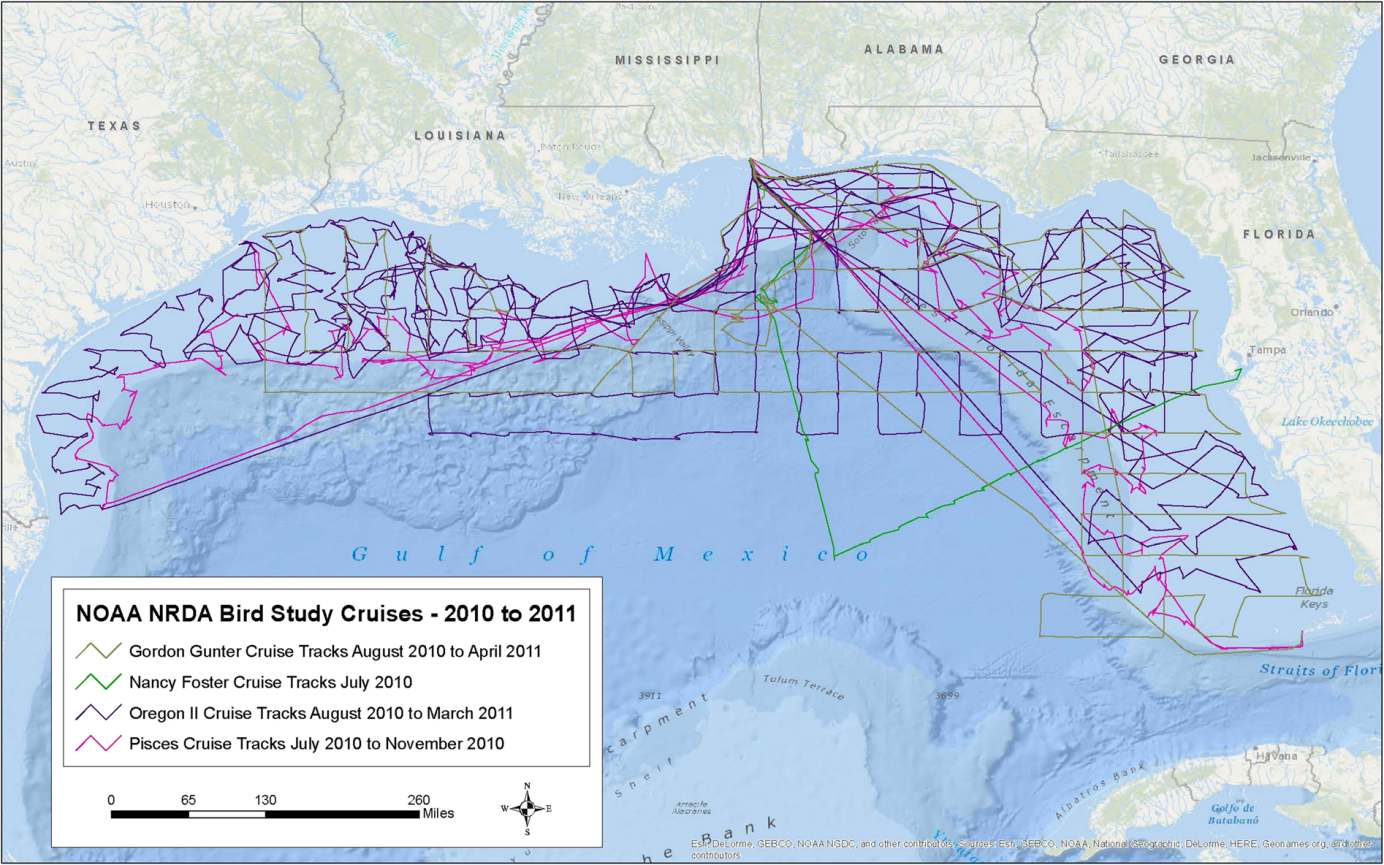
Geographic coverage of surveys conducted from July 2010 to April 2011 for marine birds in the Gulf of Mexico following the *Deepwater Horizon* blow out

Spatial coverage of surveys was extensive (Fig. 1). In general, spatial coverage by the surveys in this study was more thorough in the eastern and north-central Gulf of Mexico, nearer the *Deepwater Horizon* well head, with somewhat less coverage in the southwestern Gulf and in the deepest portions far beyond the edge of the continental shelf. Regions surveyed included both continental shelf and deep pelagic marine habitats. Continental shelves of southern, central and eastern Texas, Louisiana, Mississippi, Alabama, and west Florida were all surveyed. Shelf habitats were surveyed from just north and west of the Florida Keys to at least 96°W latitude off central Texas. Deep water, pelagic habitats surveyed included the Loop Current, associated eddies and jets, continental slopes, the Desoto Canyon region off Alabama and northwest Florida, and the Mississippi Canyon area at and immediately adjacent to the *Deepwater Horizon* well head. Geographic coverage overlapped extensively with the cumulative spill zone (Fig. 1).

From July 2010 to July 2011, observers surveyed the Gulf of Mexico’s coastal, offshore, and pelagic waters for marine birds on 285 days. Surveys were conducted throughout the year and in all seasons. Surveys were conducted every month except January, when the research ships are docked for routine annual maintenance. Months with the greatest survey effort were April and May, and July through November.

It is noteworthy that the survey effort was considerably greater in the late summer and fall. In fact, the amount of survey coverage from the NRDA study during the fall months exceeded even the combined surveys of all other previous Gulf studies of pelagic birds. A total of 27 cruise or cruise legs (at-sea deployments interrupted by one or more port calls) were conducted. The cumulative length of a particular individual cruise or cruise leg varied between 5 and 19 days (Table 1).

### Abundance and diversity of birds observed

Combining the counts from both the strip transects and point counts for all surveys, more than 23,000 individual birds were observed among all cruises, a total that included 45 different species of estuarine, coastal, offshore, and pelagic birds (Table 2). Observers sometimes made notations of terrestrial, non-aquatic bird species and other marine life that were observed during station point counts and/or transect surveys. These data are not included in this analysis.

**Table 2:** Seabird species observed in the Gulf of Mexico in waters near and adjacent to the *Deepwater Horizon* spill. Other aquatic species, such as herons and osprey, and all terrestrial species that were seen flying over the ocean are not included in this table. Nomenclature here follows A.O.U. (2011; see http://www.aou.org/checklist/north/, accessed 12 September 2011). Numbers in column two are the approximate minimum due to using the lowest number when observers reported individual birds in units of “tens” or “hundreds”

Common name	Scientific name	Approximate minimum total number recorded (transects and point count surveys combined)
Black scoter	*Melanitta americana*	1
Sea duck sp.	n/a	447
Common loon	*Gavia immer*	51
Horned grebe	*Podiceps auritus*	108
Black-capped petrel	*Pterodroma hasitata*	8
*Pterodroma* sp.	*Pterodroma* sp.	1
Cory’s shearwater	*Calonectris diomedea*	61
Great shearwater	*Puffinus gravis*	66
Sooty shearwater	*Puffinus griseus*	1
Audubon’s shearwater	*Puffinus lherminieri*	585
Shearwater sp.	*Puffinus* sp.	28
Wilson’s storm-petrel	*Oceanites oceanicus*	27
Leach’s storm-petrel	*Oceanodroma leucorhoa*	2
Band-rumped storm-petrel	*Oceanodroma castro*	35
Storm-petrel sp.	*Oceanodroma* sp.	5
White-tailed tropicbird	*Phaethon lepturus*	1
Red-billed tropicbird	*Phaethon aethereus*	5
Tropicbird species	*Phaethon* sp.	2
Magnificent frigatebird	*Fregata magnificens*	344
Masked booby	*Sula dactylatra*	35
Brown booby	*Sula leucogaster*	7
Sulid sp.	*Sula* sp.	5
Northern gannet	*Morus bassanus*	1026
Neotropic cormorant	*Phalacrocorax brasilianus*	6
Double-crested cormorant	*Phalacrocorax auritus*	100
White pelican	*Pelecanus erythrorhynchos*	1
Brown pelican	*Pelecanus occidentalis*	2693
*Phalaropus* sp.	*Phalaropus* sp.	56
Red-necked phalarope	*Phalaropus lobatus*	21
Red phalarope	*Phalaropus fulicarius*	1
Bonaparte’s gull	*Chroicocephalus philadelphia*	90
Laughing gull	*Leucophaeus atricilla*	6772
Franklin’s gull	*Leucophaeus pipixcan*	1
Ring-billed gull	*Larus delawarensis*	15
Herring gull	*Larus argentatus*	1531
Great black-backed gull	*Larus marinus*	2
Gull sp.	*Larus* sp.	141
Brown noddy	*Anous stolidus*	16
Sooty tern	*Onychoprion fuscatus*	871
Bridled tern	*Onychoprion anaethetus*	86
Sooty/bridled-type terns	*Onychoprion* sp.	36
Least tern	*Sternula antillarum*	23
Gull-billed tern	*Gelochelidon nilotica*	18
Caspian tern	*Hydroprogne caspia*	4
Black tern	*Chlidonias niger*	2323
Roseate tern	*Sterna dougallii*	2
Common tern	*Sterna hirundo*	1905
Forster’s tern	*Sterna forsteri*	31
Royal tern	*Thalasseus maximus*	1785
Sandwich tern	*Thalasseus sandvicensis*	1415
Tern sp.	Tern sp.	489
South polar skua	*Stercorarius maccormicki*	1
Skua/large dark jaeger	*Stercorarius* sp.	2
Small jaeger	*Stercorarius* sp.	10
Pomarine jaeger	*Stercorarius pomarinus*	79
Parasitic jaeger	*Stercorarius parasiticus*	18
Long-tailed jaeger	*Stercorarius longicaudus*	2
	Total species = 45+	Total individuals = 23,397

Daily relative abundance of birds detected varied extensively, mostly depending on location and season, but also due to smaller changes in survey effort among cruises and/or days. An approximate average daily abundance of seabirds detected on each individual survey cruise, and thus partially adjusted for survey effort, varied from a low of approximately 7 birds/day in November 2010 along regions of the mid-and outer continental shelf to a high of more than 580 birds/day in June 2011 within the productive near-shore, coastal waters of the northern Gulf.

### Oiled bird observations

Out of all birds observed, 1393 individual birds (6%) were assessed for signs of external oiling. At least seven birds were determined as oiled. Oiled individuals were in the Trace (five birds) and Light (2 birds) categories. Oiled species include laughing gull (5), sandwich tern (1), and sooty tern (1). Locations for visually oiled birds ranged from waters off eastern Texas to north of the Florida Keys. The vast majority of individual seabirds detected by naked eye or through binoculars during pelagic surveys, however, could not be reliably determined as oiled or not, primarily due to the limitations imposed by viewing distance.

## Discussion

### Abundance and diversity of birds observed

**Fig. 2:**
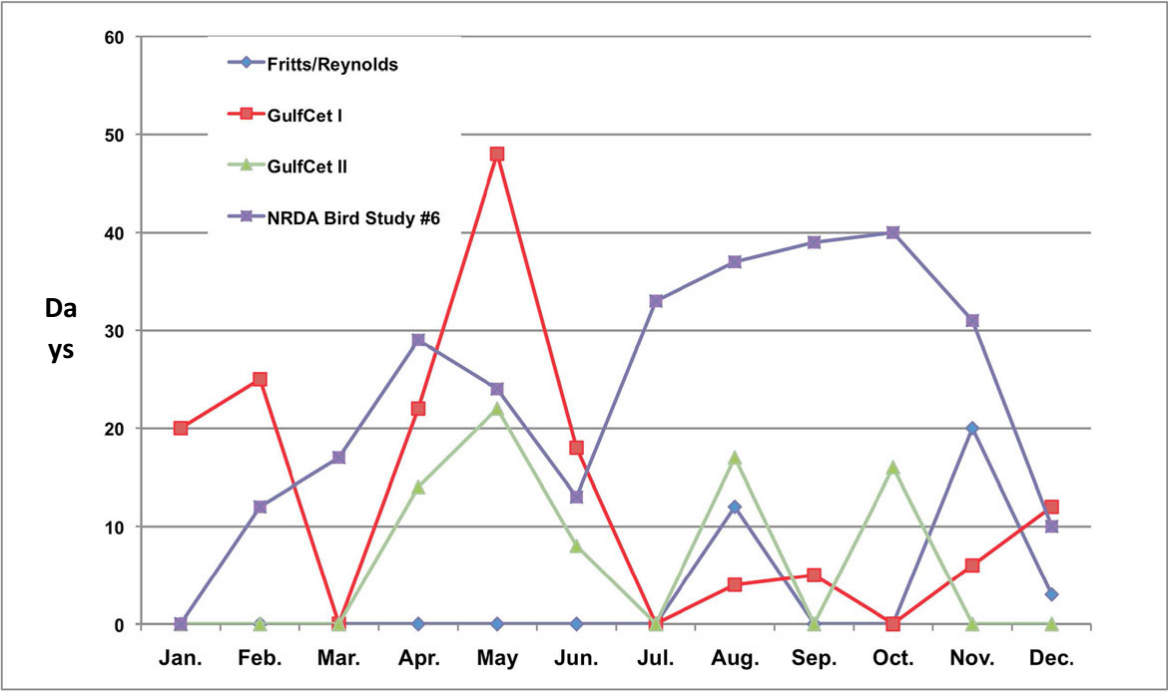
Comparison of the seasonal coverage (days surveyed during the months of the seasons) from four (4) different formal, standardized surveys of pelagic birds conducted in the Gulf of Mexico. NRDA Bird Study #6 refers to the study summarized herein. The study by Fritts and Reynolds (1981) was an aerial survey carried out in the far eastern and far western Gulf. The GulfCet I and GulfCet II surveys (April 1992 to June 1994), in the northwestern and northeastern Gulf, were aimed primarily at research on marine mammals and their habitats, but bird surveys were also conducted opportunistically (e.g., see Davis and Fargion 1996)

At least 23,377 individual birds were observed among all cruises and using both survey techniques. That total represents the minimum detected because observers’ tallies of, e.g., “hundreds” were rounded down to the lowest possible figure. This total is noteworthy considering it is almost three times more birds than recorded in the previous GulfCet II pelagic surveys (8507 individual birds; 17 April–31 July 1996), greater than eight times more birds than recorded in the GulfCet I pelagic surveys (2692 individual birds), and better than eleven times more birds than recorded during the Fritts and Reynolds (1981) aerial surveys (1946 individual birds) (Fig. 2). As a result of greater number of survey days, the total numbers of individual birds recorded during NRDA Bird Study #6 exceed the combined totals of all previous standardized, formal seabird studies ever conducted in the Gulf of Mexico.

Differences in the numbers of total birds recorded among the different seabird survey programs, however, do not necessarily reflect changes in seabird abundance across time or space. Rather, the disparity in aggregate numbers highlight the paucity of previous effort devoted to seabird surveys in the Gulf of Mexico prior to the 2010 *DWH* spill. In addition, the various earlier surveys used such different methodologies that most are not comparable using measures of normalized effort, such as expressing the number of birds observed per unit area surveyed. Previous surveys of marine birds in the Gulf also lack some of the key meta-data that might enable more robust comparisons to NRDA Bird Study #6 data.

An exception to limitations on comparison is a new survey of marine birds now underway, the Gulf of Mexico Marine Assessment Program for Protected Species (GoMMAPPS; https://www.boem.gov/GOMMAPPS/). Begun in early 2017 and adopting the same counting methodology as NRDA Bird Study #6, it uses consistent methodology between the two studies (Table 3). When cruises had an identical purpose (NOAA spring plankton survey), were carried out in the same region of the western Gulf, used the same cruise track, and were even conducted during similar dates, the results were broadly similar. Slight differences observed may be explained by different oceanographic conditions prevailing in the Gulf between the study timeframes, or depressed bird abundance in the earlier study after the spill.

**Table 3:** Seabird densities from NRDA Bird Study 6 compared to a later study also using line transect survey methodology. GoMMAPPS surveys (Gulf of Mexico Marine Assessment Program for Protected Species) started in 2017

	NRDA Bird Study 6	GoMMAPPS
Survey dates	13–27 May 2011	16–30 May 2017
Total distance actively surveyed (km)	1128	1792
Total hours surveyed	77.33	99.55
Birds observed on transect	305	649
Mean density (birds per km^2^)	0.27	0.36
Minimum daily density (birds per km^2^)	0.01	0.04
Maximum daily density (birds per km^2^)	1.41	2.92
Species richness	7	22

Study observations largely matched the expected species composition based on a literature review that occurred prior to the study. Of the 45 different species of estuarine, coastal, offshore, and pelagic birds recorded during this study, 42 of these species had been identified in survey planning (Boyce et al. 2010) and the corresponding instruction manual (Haney 2010). These 42 species were expected to occur among all calendar seasons. Species expected based on the literature but that were not observed included: pied-billed grebe (*Podilymbus podiceps*), yellow-nosed albatross (*Thalassarche chlororhynchos*), Manx shearwater (*Puffinus puffinus*), red-footed booby (*Sula sula*), Sabine’s gull (*Xema sabini*), arctic tern (*Sterna paradisaea*), and black skimmer (*Rhynchops niger*). However, the pied-billed grebe and black skimmer are not typically seen in the deep, pelagic marine waters of the Gulf of Mexico; and the yellow-nosed albatross (*Thalassarche chlororhynchos*), red-footed booby (*Sula sula*), Sabine’s gull (*Xema sabini*), and arctic tern (*Sterna paradisaea*) are exceedingly uncommon in the northern Gulf of Mexico (e.g., species accounts in Rodewald 2015).

Manx shearwater is known to have occurred in the Gulf during the time of the spill, however, because dead individuals of this species were reported in the Bird Impact Data from the DOI-ERDC NRDA Database, dated 12 May 2011 (see http://www.fws.gov/home/dhoilspill/collectionreports.html; accessed 13 September 2011). Conversely, black scoter (*Melanitta americana*), neotropic cormorant (*Phalacrocorax brasilianus*), and white pelican (*Pelecanus erythrorhynchos*) were not anticipated to be observed far offshore based on the literature, but were nevertheless found there.

Overall, and despite some notable seasonal differences in the composition of the marine bird community in the Gulf of Mexico, the annualized composition of seabirds recorded in this study tended to be strongly dominated by larids. More than 75% of all individual seabirds recorded across the study were gulls, terns, and skuas. The next most dominant group was the pelicaniformes (boobies, pelicans, etc.), making up 18% of all individual seabirds recorded. The procellariiformes, or tube-nosed birds (e.g., shearwaters, storm-petrels), comprised less than 4% of all seabirds detected during the study.

### Abundance

Daily relative abundance of birds varied, depending on location and season. Average daily abundance for seabirds per individual cruise varied from a low of approximately 7 birds/day in November 2010 along regions of the mid-and outer continental shelf to a high of more than 580 birds/day in June 2011 within the near-shore, coastal waters of the northern Gulf.

As expected for the Gulf of Mexico, there were locations and seasons where pelagic densities were extremely low. Low seabird densities are characteristic of nutrient-poor tropical or subtropical seas like those in the offshore Gulf (e.g., Zakkak et al. 2013; de Boer et al. 2014). Observers might on occasion go for an entire day without seeing any birds, especially far offshore on the middle and outer continental shelf, or in very deep waters far from any biological hotspots associated with edges of the Loop Current or other prominent oceanographic features. Based on the collective data, particular locations and times were considered to be low in abundance if less than 20 birds were observed in a day. Conversely, based on the collective data, specific locations and times were considered to be particularly high in abundance when more than 200 birds were observed in a day, which often coincided with fishing trawl activity.

### Oiling rates

Out of the grand total, 1393 birds were assessed for external oiling, and seven birds were positively determined to be trace or lightly oiled. The efficacy of visually determining oiling rates was likely quite low due to the nature of these surveys, i.e., examining birds at a distance while in motion. It is extremely difficult to identify birds with trace or light oiling from a moving ship. Both the Trustees and Haney et al. (2014) independently estimated the number of oiled birds to be much greater using methods applied to these abundance data and surface oil information from the DWH spill (Haney et al. 2014; Industrial Economics, Inc. 2015).

## Conclusion

This extensive 13-month survey documented the variety and numbers of birds offshore in the Gulf of Mexico coastline. In total, more than 23,000 individual birds were observed among all cruises and techniques. This total was far greater than all previous standardized, formal seabird studies conducted in the Gulf of Mexico. Forty-five different species of estuarine, coastal, offshore, and pelagic birds were observed. Overall, and despite some notable seasonal differences in composition of the marine bird community in the Gulf of Mexico, seabirds in this Gulf study were strongly dominated by larids, followed by pelicaniformes, and then procellariiformes.

Daily relative abundance of birds varied depending on location and season. Average daily abundance Gulf-wide for seabirds varied from a low of approximately 7 birds/day to a high of more than 580 birds/day. However, only seven birds were observed to be visual oiled largely because the nature of these surveys prevented detailed examination of potentially oiled birds, i.e., limitations imposed by observing birds at a distance while the ship was in motion. It is extremely difficult to identify all or most birds with trace or light oiling under such viewing conditions.
